# Lesion Characteristics Associated with Loss of Primary Patency After Endovascular Therapy for Common Femoral Artery Lesions

**DOI:** 10.1007/s00270-022-03343-4

**Published:** 2023-01-10

**Authors:** Yasutaka Yamauchi, Mitsuyoshi Takahara, Yo Iwata, Kenji Suzuki, Naoki Fujimura, Terutoshi Yamaoka, Akira Miyamoto, Tatsuya Nakama, On Behalf of the CAULIFLOWER Study Investigators

**Affiliations:** 1 Cardiovascular Center, Takatsu General Hospital, Kanagawa, Japan; 2 Department of Diabetes Care Medicine, Osaka University Graduate School of Medicine, Osaka, Japan; 3 Department of Cardiology, Heart and Vascular Institute, Funabashi Municipal Medical Center, Chiba, Japan; 4 Department of Cardiology, Tokyo Saiseikai Central Hospital, Tokyo, Japan; 5 Department of Vascular Surgery, Tokyo Saiseikai Central Hospital, Tokyo, Japan; 6 Department of Vascular Surgery, Matsuyama Red Cross Hospital, Ehime, Japan; 7 Department of Cardiology, Tokyo Bay Medical Center, Chiba, Japan

**Keywords:** Common femoral artery, Endovascular therapy, Primary patency

## Abstract

**Purpose:**

To identify lesion characteristics associated with restenosis after endovascular therapy (EVT) for common femoral artery (CFA) lesions in patients with peripheral artery disease (PAD) in real-world practice.

**Materials and Methods:**

We included 751 Japanese patients with PAD who underwent CFA EVT. Data were from a large-scale retrospective multicenter registry study. The association of lesion characteristics with the risk of restenosis was investigated with the Cox proportional hazards regression model.

**Results:**

Lesions extended to the external iliac artery in 10.0% of patients, were isolated in the CFA in 59.9%, and involved the bifurcation in 30.1%. Chronic total occlusion was noted in 21.1%, and 99% stenosis, in 19.9%. Among the limbs with CFA lesions, 16.4% had a history of CFA EVT. Mean total lesion length was 32 ± 15 mm, and reference vessel diameter, 7.3 ± 1.4 mm. Plain old balloon angioplasty, drug-coated balloon angioplasty, and stent implantation were performed in 56.3, 23.2, and 20.5% of patients, respectively. The mean follow-up period was 10.4 ± 9.5 months. Rates of freedom from restenosis and reintervention at 1 year were 78.2 and 86.6%, respectively. Lesion characteristics independently associated with restenosis were history of CFA EVT, reference vessel diameter less than 6 mm, and lesion length greater than or equal to 50 mm; adjusted hazard ratios were 1.63 (*P* = 0.007), 1.93 (*P* = 0.006), and 1.71 (*P* = 0.018), respectively.

**Conclusion:**

History of CFA EVT, smaller reference vessel diameter, and longer lesion length are independent risk factors for restenosis after CFA EVT.

**Level of Evidence:**

Level 3.

**Supplementary Information:**

The online version contains supplementary material available at 10.1007/s00270-022-03343-4.

## Introduction

Guidelines recommend thromboendarterectomy (TEA) as the first-line revascularization approach for common femoral artery (CFA) lesions because it achieves good long-term patency [1–4]. However, as a result of the rapid developments in endovascular techniques in recent decades, endovascular therapy (EVT) is now viewed as an alternative interventional modality for CFA lesions [5, 6].

In 2017, Gouëffic et al. [7] reported the first randomized controlled trial (RCT) that compared TEA and CFA stenting; CFA stenting showed comparable durability and a lower complication rate than TEA. Although some reports indicate that EVT is equivalent to TEA in terms of patency, others indicate that it is still not as good as TEA [1–4]. On the other hand, EVT may be superior to TEA in terms of safety, promoting a shift from TEA to EVT [7, 8].

To identify lesion characteristics associated with patency after CFA EVT, a large-scale study with periodical assessment of patency is required. Therefore, by analyzing data from the *common femoral artery revascularization; investigation of the best strategy from the multicenter retrospective real-world registry* (cauliflower) registry database [8], the current study aimed to identify lesion characteristics associated with restenosis risk after EVT for CFA lesions in patients with symptomatic peripheral artery disease (PAD).

## Materials and Methods

### Patients and Lesions

To clarify clinical outcomes, the CAULIFLOWER study enrolled all consecutive patients undergoing CFA revascularization. In the present study, we analyzed data from the CAULIFLOWER study to identify independent lesion characteristics associated with restenosis in Japanese patients after CFA EVT. We included data on 751 consecutive patients with symptomatic PAD (Rutherford category 2–5) who underwent EVT for CFA lesions between January 2018 and June 2020 at 52 centers. Patients with non-atherosclerotic lesions, in-stent restenosis, previous failed EVT at other facilities, restenosis after TEA, or restenosis of a bypass anastomosis were excluded from the study. The classifications of CFA lesions were as follows: type I, lesions extending to the external iliac artery; type II, isolated CFA lesions; type III, lesions located at the CFA and its bifurcation; and type IV, restenosis of a bypass anastomosis [9]. As mentioned above, type IV lesions were not included in the present study. Calcification was evaluated by computed tomography and angiography as the presence or absence of nodular calcification, which was defined as coral reef-like calcification protruding into the lumen [8]. Angiographic or imaging depiction of CFA lesion types was so far lacking for a study focused on lesion predictors. In this study, the following lesion characteristics were evaluated in angiographic images: degree of stenosis, presence of nodular calcification, reference vessel diameter, and lesion length.

The study was approved by the ethics committee of each participating institution. For this type of study, informed consent is not required, but patients were able to opt out of the study.

### Endovascular Therapy Procedure

EVT was performed mainly by a 5–7 Fr system. During the procedure, we infused 5000 U of heparin and gave additional doses of heparin as needed to maintain an activated clotting time of 200 s or longer. The lesion was crossed with a 0.014- or 0.018-inch guidewire. After successful crossing with the guidewire, balloon dilation and stenting of the lesion were performed by percutaneous old balloon angioplasty (POBA), drug-coated balloon (DCB) angioplasty, or stent implantation with the optimal device. Patients who underwent DCB angioplasty or stent implantation were treated with dual antiplatelet therapy with aspirin and thienopyridine for at least one month after EVT. All patients received at least one antiplatelet drug indefinitely. The treating operators selected the EVT strategy, guidewires, balloons, stents, and antiplatelet therapy.

### Outcome Measure

The outcome measure was restenosis after EVT, which was defined as peak systolic velocity ratio greater than or equal to 2.8 on duplex ultrasound or greater than or equal to 50% restenosis on follow-up with computed tomography or angiography. As recommended in the society of vascular surgery report [10], the frequency of imaging evaluation was every 3 months during the first year and then every 6 months or annually.

### Statistical Analysis

Data on baseline characteristics are presented as means ± standard deviation (SD) for continuous variables and frequency and percentage for categorical variables. *P* values of less than 0.05 were considered statistically significant, and 95% confidence intervals (95% CIs) were reported where appropriate. The rates of freedom from restenosis and reintervention were estimated by the Kaplan–Meier method. The association of lesion characteristics with the risk of restenosis was investigated with the Cox proportional hazards regression model. The following lesion characteristics were entered as the explanatory variables: history of CFA EVT, CFA lesion type, stenosis, presence of nodular calcification, reference vessel diameter less than 6 mm, and lesion length greater than or equal to 50 mm. The association was adjusted for endovascular device, including scoring balloon, non-compliant balloon, stent, drug-coated balloon, and intravascular ultrasound, because the device was expected to have a large influence on outcome. Statistical analyses were performed with R version 4.1.1 (R Development Core Team, Vienna, Austria; RRID:SCR_001905).

## Results

Baseline characteristics are shown in Table 1. The most common Rutherford class was 3 (53.9% of patients), and the most common CFA lesion was type II (59.9% of patients). Subtotal occlusion (i.e., 99% occlusion) and chronic total occlusion were noted in 19.9 and 21.1% of patients, respectively. POBA was used for revascularization in 56.3% of patients. Type I, II, and III lesions had a mean length of 34.7 ± 15.9 mm, 29.5 ± 12.9 mm, and 37.4 ± 17.0 mm, respectively; the proportion of lesions with a length of 50 mm or longer was 11.3%, 7.3%, and 16.6% in type I, II, and III, respectively.

**Table 1:** Baseline characteristics of the study population (*N* = 751)

Characteristic	Frequency as n (%) or mean ± standard deviation
Male sex	551 (73.4%)
Age, year	74 ± 9
Non-ambulatory	73 (9.7%)
Smoker	497 (66.2%)
Type 2 diabetes	437 (58.2%)
Chronic kidney disease	
No	288 (38.3%)
Yes, but no dialysis	215 (28.6%)
Yes, and on dialysis	248 (33.0%)
Cerebrovascular disease	148 (19.7%)
Coronary artery disease	390 (51.9%)
Chronic heart failure	139 (18.5%)
Atrial fibrillation	114 (15.2%)
Rutherford classification	
Category 2	154 (20.5%)
Category 3	405 (53.9%)
Category 4	94 (12.5%)
Category 5	98 (13.0%)
Ankle brachial index^a^	0.55 ± 0.28
History of CFA endovascular therapy	123 (16.4%)
CFA lesion type	
Type I	75 (10.0%)
Type II	450 (59.9%)
Type III	226 (30.1%)
CFA stenosis^a^	
50–90%	443 (59.1%)
99% (subtotal occlusion)	149 (19.9%)
100% (chronic total occlusion)	158 (21.1%)
Nodular calcification	543 (72.3%)
Reference vessel diameter, mm^a^	7.3 ± 1.4
< 6 mm	66 (8.8%)
Lesion length, mm^a^	32 ± 15
≥ 50 mm	77 (10.4%)
Scoring balloon used for dilatation	240 (32.0%)
Non-compliant balloon used for dilatation	316 (42.1%)
Revascularization strategy	
Plain old balloon angioplasty	423 (56.3%)
Stent implantation	154 (20.5%)
Drug-coated balloon treatment	174 (23.2%)
Intravascular ultrasound use	484 (64.4%)
Additional AI revascularization	214 (28.5%)
Additional SFA revascularization	309 (41.3%)

AI, aortoiliac; CFA, common femoral artery; SFA, superficial femoral artery

^a^Data were missing, as follows: ankle brachial index, 25 patients (3.3%); common femoral artery stenosis, 1 patient (0.1%); reference vessel diameter, 2 patients (0.3%); lesion length, 13 patients (1.7%); and additional superior femoral artery revascularization, 2 patients (0.3%)

**Fig. 1:**
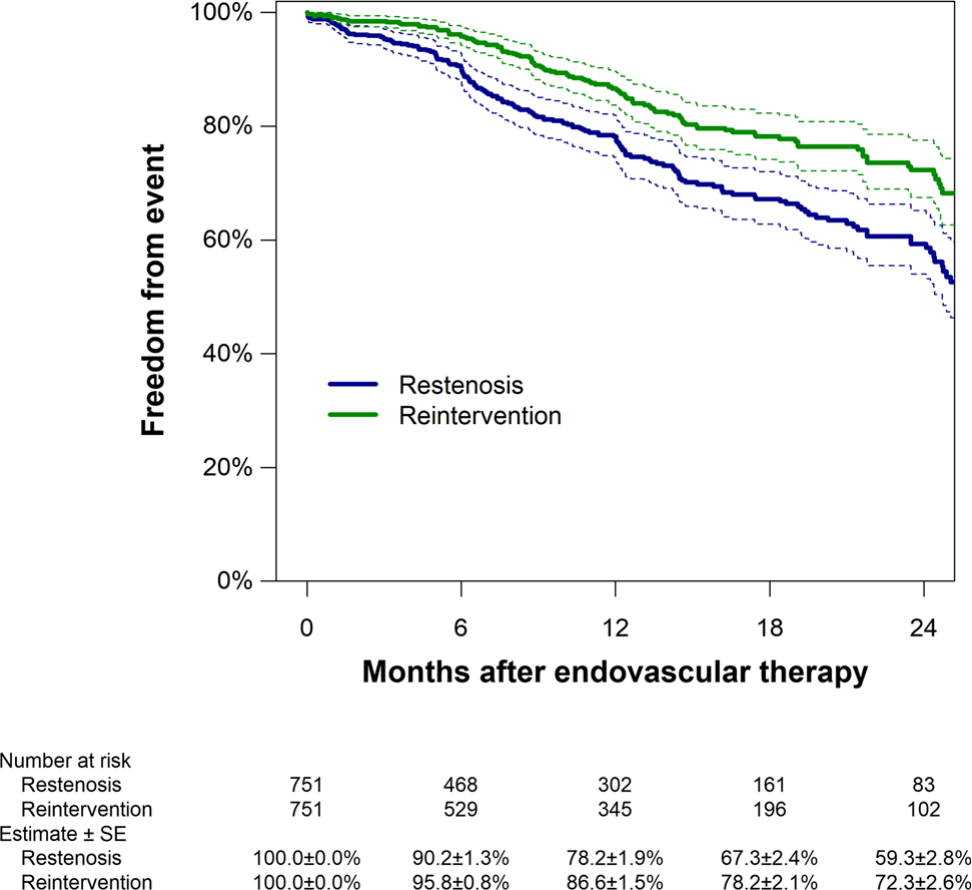
Freedom from restenosis and reintervention in the overall population. Dotted lines indicate 95% confidence intervals. SE, standard error

During a mean follow-up period of 10.4 ± 9.5 months, restenosis occurred in 174 patients; the remaining 577 patients were treated as censored cases. Of these 577 patients, 80 died without experiencing restenosis. Patients who were alive at 1 year accounted for 36.2% (*n* = 272) of the overall population; Supplementary Table S1 shows the baseline characteristics of the whole population according to whether or not patients were alive at 1 year. The 1-year rates of freedom from restenosis and reintervention were 78.2% (95% confidence interval, 74.6–81.9%) and 86.6% (83.7–89.7%), respectively (Fig. 1).

**Fig. 2:**
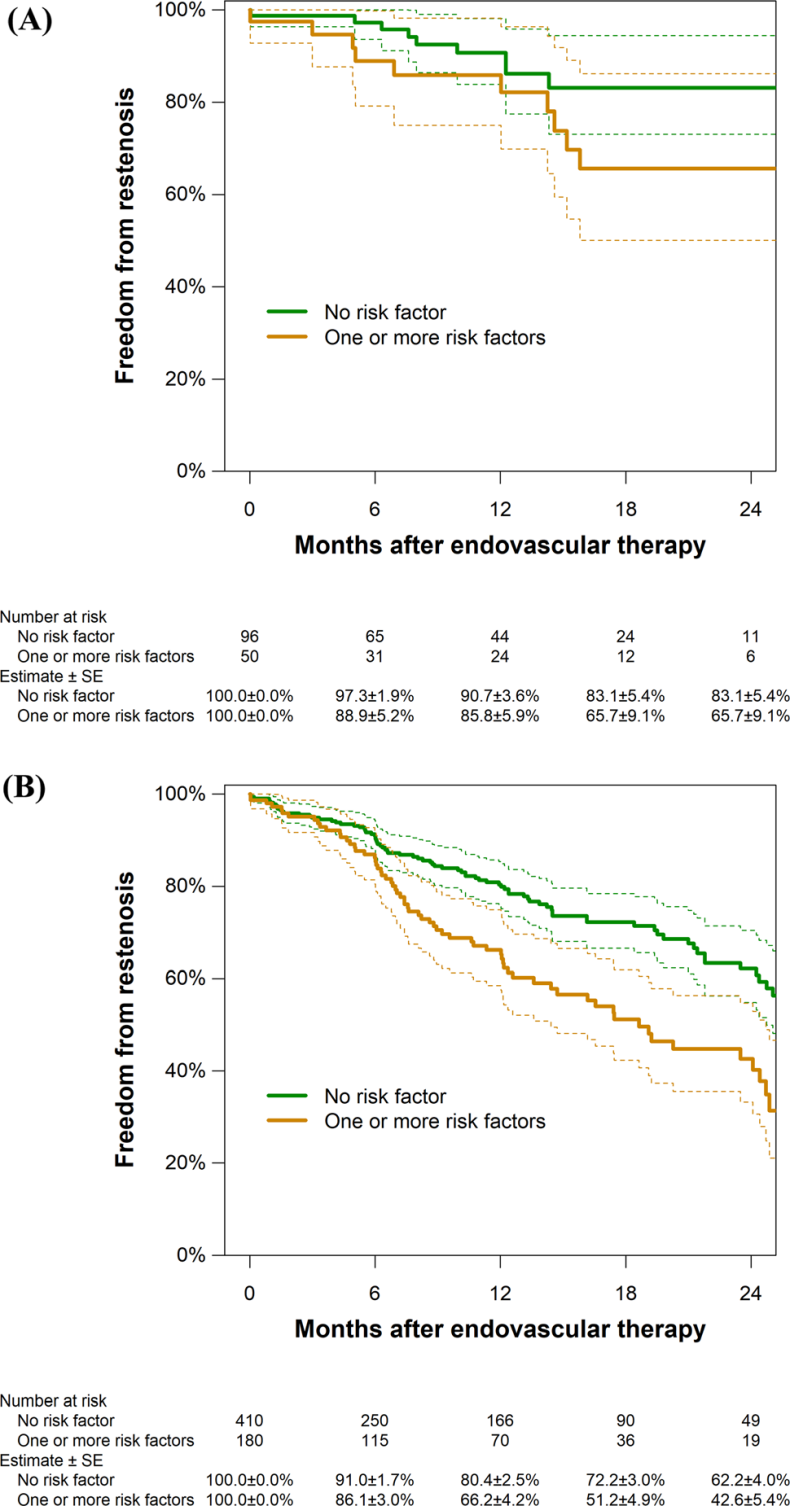
Freedom from restenosis by the presence of risk factors in the groups with (**A**) and without stent implantation (**B**). Dotted lines indicate 95% confidence intervals. Risk factors were (1) history of common femoral artery (CFA) revascularization, (2) small reference vessel diameter (< 6 mm), and (3) long CFA lesion (≥ 50 mm), SE, standard error

Table 2 shows the association of baseline characteristics with the restenosis risk. Stent implantation was the only procedure-related risk factor, and all other risk factors were related to lesion characteristics. Stent implantation was inversely associated with an increased risk of restenosis, and history of CFA EVT, reference vessel diameter less than 6 mm, and lesion length greater than or equal to 50 mm were independently associated with a higher risk. No clinical factors or additional revascularization for aortoiliac or superficial femoral artery (SFA) lesions were significantly associated with the risk of restenosis (Supplementary Table S2). When participants were stratified into groups with and without stent implantation, patients with one or more of the identified risk factors had a lower rate of freedom from restenosis, regardless of stent implantation (Fig. 2A, B): The HR was not significantly different between the groups with (2.07; 95% CI, 0.86–4.98) and without a stent (1.86; 95% CI 1.35–2.57; *P* for interaction = 0.82).

**Table 2:** Association of lesion characteristics with restenosis risk

	Crude hazard ratio (95% confidence interval)	Adjusted hazard ratio (95% confidence interval)
History of CFA endovascular therapy	1.62 [1.15–2.28] (*P* = 0.006)	1.73 [1.21–2.49] (*P* = 0.003)
CFA lesion type (vs Type II)		
Type I	1.12 [0.67–1.88] (*P* = 0.66)	1.25 [0.69–2.25] (*P* = 0.47)
Type III	1.19 [0.86–1.65] (*P* = 0.30)	1.15 [0.81–1.63] (*P* = 0.42)
CFA stenosis (vs 50–90%)		
99% (subtotal occlusion)	1.27 [0.87–1.87] (*P* = 0.21)	1.34 [0.90–2.00] (*P* = 0.15)
100% (chronic total occlusion)	1.29 [0.89–1.87] (*P* = 0.18)	1.44 [0.95–2.17] (*P* = 0.085)
Nodular calcification	1.14 [0.80–1.61] (* P* = 0.47)	1.22 [0.83–1.78] (* P* = 0.31)
Reference vessel diameter < 6 mm	1.92 [1.23–3.00] (*P* = 0.004)	1.98 [1.23–3.17] (*P* = 0.005)
Lesion length ≥ 50 mm	1.77 [1.18–2.66] (*P* = 0.006)	1.68 [1.08–2.61] (*P* = 0.022)
Scoring balloon used for dilatation	1.10 [0.81–1.50] (*P* = 0.53)	1.07 [0.72–1.60] (*P* = 0.73)
Non-compliant balloon used for dilatation	0.94 [0.69–1.28] (*P* = 0.68)	0.92 [0.62–1.36] (*P* = 0.66)
Revascularization strategy (vs plain old balloon angioplasty)		
Stent implantation	0.52 [0.33–0.83] (*P* = 0.006)	0.46 [0.28–0.75] (*P* = 0.002)
Drug-coated balloon treatment	1.13 [0.78–1.65] (*P* = 0.52)	1.13 [0.77–1.67] (*P* = 0.54)
Intravascular ultrasound use	1.11 [0.82–1.51] (*P* = 0.51)	1.05 [0.75–1.45] (*P* = 0.79)

CFA, common femoral artery

## Discussion

This subgroup analysis of a multicenter retrospective study population showed that the estimated 1-year restenosis rate after CFA EVT was low (21.8%) and the 1-year primary patency rate was high (78.2%). The independent lesion characteristics associated with loss of primary patency were history of CFA EVT, small reference vessel diameter (< 6 mm), and long lesion (≥ 50 mm). Rabellino et al. [11] advocated for the inclusion of new CFA lesion distribution, chronic total occlusion, and severe calcification in the risk classification; however, this study included only a small number of patients and did not analyze factors such as lesion length and vessel diameter and did not perform multivariate analysis of factors. The strength of the CAULIFLOWER registry was that we identified factors of lesion characteristics independently associated with loss of primary patency in CFA EVT [8].

Little information is available on risk factors for restenosis in CFA EVT, but we hypothesize that findings in the iliac artery and SFA may also apply to CFA EVT. The previous studies reported that a small vessel diameter was a predictor of poor patency in patients with aortoiliac lesions [12] and lesions in the femoropopliteal region [13, 14]. These results are in line with our finding that small vessel diameter is a predictor of future restenosis after CFA EVT.

Earlier results showed that a long lesion does not predict primary patency in patients with aortoiliac lesions [12, 15] and CFA lesions [16], but in the current study, we found that a longer CFA lesion (≥ 50 mm) was significantly associated with loss of primary patency. The frequency of stent use in this study was not high, so lesion length may have affected results. In other words, it is important to consider that the different treatment strategies used do not allow direct comparisons to be made with results of other studies [17, 18]. Another study obtained the same result in the femoropopliteal region: Soga et al. [19] included six items in the risk stratification of vessel patency and mortality after self-expandable nitinol stenting for superficial femoral artery disease and found that a lesion length greater than 150 mm was the most important attribute. Previous studies showed that outflow lesions have a large effect on patency after aortoiliac stent placement, especially in the case of CFA lesions located immediately after the outflow tract. Timaran et al. [20] reported that CFA lesions with poor run off (ad hoc committee on reporting standards score > 5 [21]) were independent predictors for primary patency after aortoiliac EVT. However, in the present study, type I lesions were not significantly associated with patency rate. The reason for this discrepancy is not clear, but the type of lesion may not have affected patency rate after aortoiliac stenting because aortoiliac stenting has extremely good clinical outcomes [15, 22]. Our findings indicate that longer lesion length is more important for restenosis risk than lesion localization and that longer lesion length is associated with a higher risk of restenosis in the CFA only in isolated cases, whereas shorter lesion length is associated with a lower risk of restenosis even if the lesion extends to the external iliac artery or the bifurcation. Thus, lesion length may be a negative predictor of restenosis after EVT. TEA for type I lesions is expected to be more invasive because it requires larger surgical wounds. From this perspective, EVT may be appropriate for polyvascular disease, including CFA lesions.

In patients who underwent aortoiliac angioplasty or stenting for PAD, Gheini et al. [16] showed that restenosis was significantly associated with the vessel that underwent EVT and the EVT technique. CFA often has unique characteristics, such as coral reef-like calcification protrusions into the lumen, and this severe calcification cannot be removed when using only conventional EVT, i.e., POBA. Currently, POBA has a poor patency rate, and EVT has to be repeated in many patients [8]. Restenosis lesions are often characterized by poor patency because of their calcification characteristics. A history of revascularization and the presence of many comorbidities and complex lesions may be associated with a high restenosis rate after POBA. Therefore, we believe that a more effective treatment than POBA is needed.

Repeated EVT may require more widespread target vessel revascularization than TEA because target vessel embolization or dissection is likely the reason for restenosis. Even if CFA restenosis lesions are treated with TEA, the clinical results of the additional TEA might be poor. Therefore, when treating patients with CFA lesions, TEA may be the preferred treatment approach in patients with the risk factors identified in this study. Moreover, it is necessary to fully consider the patient’s background and tolerance of surgery. Repeated EVT increases the physical burden on the patient but also increases the cost of medical care.

Because we expected the endovascular device used to have a large influence on clinical outcome, we examined the interaction effect of lesion characteristics with and without stent implantation. We found no difference between the groups, showing that lesion factors affect risk independent of the use of a stent (Fig. 2A, B).

Supplementary Table S1 shows which patients were lost to follow-up. We compared background characteristics of patients who were alive at the 1-year follow-up and those who were not (Supplementary Table S1). In many of the patients who were lost to follow-up, the reference vessel diameter was large or a stent was used. We suggest that patients with a patent vessel who have no recurrence of symptoms may be more likely to be lost to follow-up. If this was the case, the loss of patients may have resulted in the patency rate being underestimated.

## Limitations

This study has several limitations. First, it included only Japanese patients, and we do not know whether similar results would be obtained in other ethnic groups where these devices are frequently used. Second, patency was not assessed by a central laboratory; although this might have potentially undermined the reliability of restenosis evaluations, each participating site had experience with conducting pivotal clinical trials, which was considered likely to minimize variation in assessment. Third, the study did not collect data on how individual operators evaluated lesion diameter or length or how they selected the diameter and length of the stents or balloons. Fourth, we did not collect data on whether a self-expandable or balloon-expandable stent was used. Fifth, the rate of stent use was low. Sixth, we did not analyze detailed data on calcification other than the presence of nodular calcification. Seventh, no further details on the outflow situation were available. Eighth, atherectomy and newer specifically designed open cell stents were not used during CFA EVT in the current study population. These devices could change clinical outcomes and potentially affect the results. And last, the rate of continuous follow-up up to 1 year was low.

## Conclusion

The lesion characteristics history of CFA EVT, smaller vessel diameter (< 6 mm), and longer lesion (≥ 50 mm) are independent risk factors for restenosis after EVT for CFA lesions.

## Supplementary Information

Below is the link to the electronic supplementary material.

Supplementary file1 (DOCX 24 KB)

## Data Availability

Because of the nature of this research, participants of this study did not agree for their data to be shared publicly, so supporting data are not available.

## Material Availability

All the materials are original, and we did not need permission to reproduce material from other sources.
