# Supplementary material for: Lesion Characteristics Associated with Loss of Primary Patency After Endovascular Therapy for Common Femoral Artery Lesions
Source: Cardiovasc Intervent Radiol. 2023 Jan 10;46(3):310–6. doi: 10.1007/s00270-022-03343-4 (PMC10014791; doi:10.1007/s00270-022-03343-4)
Supplement: Supplementary file 1 — Supplementary file1 (DOCX 24 KB) [file 270_2022_3343_MOESM1_ESM.docx]

**Supplementary Table 1. Baseline characteristics in patients (N = 751)** **censored as alive or not alive at 1 year after endovascular therapy for common femoral artery lesions**

|  | Censored alive at 1 year | |  |
| --- | --- | --- | --- |
|  | Yes | No | P value |
|  | (n = 272) | (n = 479) |  |
| Male sex, n (%) | 191 (70.2%) | 360 (75.2%) | 0.17 |
| Age, mean ± SD, y | 75 ± 8 | 74 ± 9 | 0.71 |
| Non-ambulatory, n (%) | 29 (10.7%) | 44 (9.2%) | 0.60 |
| Smoker, n (%) | 153 (56.2%) | 344 (71.8%) | < 0.001 |
| Type 2 diabetes, n (%) | 157 (57.7%) | 280 (58.5%) | 0.91 |
| Chronic kidney disease, n (%) |  |  | 0.12 |
| No | 107 (39.3%) | 181 (37.8%) |  |
| Yes, but no dialysis | 87 (32.0%) | 128 (26.7%) |  |
| Yes, and on dialysis | 78 (28.7%) | 170 (35.5%) |  |
| Cerebrovascular disease, n (%) | 68 (25.0%) | 80 (16.7%) | 0.008 |
| Coronary artery disease, n (%) | 129 (47.4%) | 261 (54.5%) | 0.074 |
| Chronic heart failure, n (%) | 39 (14.3%) | 100 (20.9%) | 0.034 |
| Atrial fibrillation , n (%) | 35 (12.9%) | 79 (16.5%) | 0.22 |
| Rutherford classification, n (%) |  |  | 0.56 |
| Category 2 | 59 (21.7%) | 95 (19.8%) |  |
| Category 3 | 138 (50.7%) | 267 (55.7%) |  |
| Category 4 | 35 (12.9%) | 59 (12.3%) |  |
| Category 5 | 40 (14.7%) | 58 (12.1%) |  |
| Ankle brachial index mean ± SD | 0.59 ± 0.27 | 0.53 ± 0.29 | 0.009 |
| Missing data, n (%) | 14 (5.1%) | 11 (2.3%) | 0.060 |
| History of CFA endovascular therapy, n (%) | 36 (13.2%) | 87 (18.2%) | 0.099 |
| CFA lesion type, n (%) |  |  | 0.15 |
| Type I | 29 (10.7%) | 46 (9.6%) |  |
| Type II | 173 (63.6%) | 277 (57.8%) |  |
| Type III | 70 (25.7%) | 156 (32.6%) |  |
| CFA stenosis, n (%) |  |  | 0.30 |
| 50% to 90% | 150 (55.4%) | 293 (61.2%) |  |
| 99% (subtotal occlusion) | 59 (21.8%) | 90 (18.8%) |  |
| 100% (chronic total occlusion) | 62 (22.9%) | 96 (20.0%) |  |
| Missing data | 1 (0.4%) | 0 (0.0%) | 0.77 |
| Nodular calcification*, n (%) | 197 (72.4%) | 346 (72.2%) | 1.00 |
| Reference vessel diameter, mean ± SD, mm** | 7.5 ± 1.5 | 7.2 ± 1.3 | 0.001 |
| < 6 mm, n (%) | 24 (8.8%) | 42 (8.8%) | 1.00 |
| Missing data, n (%) | 0 (0.0%) | 2 (0.4%) | 0.74 |
| Lesion length, mm | 32 ± 14 | 33 ± 16 | 0.37 |
| ≥ 50 mm, n (%) | 20 (7.6%) | 57 (12.0%) | 0.077 |
| Missing data, n (%) | 8 (2.9%) | 5 (1.0%) | 0.10 |
| Scoring balloon used for dilatation, n (%) | 77 (28.3%) | 163 (34.0%) | 0.12 |
| Non-compliant balloon used for dilatation, n (%) | 126 (46.3%) | 190 (39.7%) | 0.089 |
| Revascularization strategy, n (%) |  |  | < 0.001 |
| Plain old balloon angioplasty | 132 (48.5%) | 291 (60.8%) |  |
| Stent implantation | 56 (20.6%) | 98 (20.5%) |  |
| Drug-coated balloon treatment | 84 (30.9%) | 90 (18.8%) |  |
| Intravascular ultrasound use, n (%) | 204 (75.0%) | 280 (58.5%) | < 0.001 |
| Additional AI revascularization, n (%) | 74 (27.2%) | 140 (29.2%) | 0.61 |
| Additional SFA revascularization, n (%) | 106 (39.0%) | 203 (42.6%) | 0.38 |
| Missing data, n (%) | 0 (0.0%) | 2 (0.4%) | 0.74 |

Intergroup differences were tested by Welch’s *t* test for continuous variables and the chi-squared test for discrete variables.

*Nodular calcifications were defined as coral reef-like calcifications protruding into the lumen

AI, aortoiliac CFA, common femoral artery; SFA, superficial femoral artery

**Supplementary Table 2. Association of baseline characteristics with restenosis risk in patients (N = 751)** **who underwent endovascular therapy for common femoral artery lesions**

|  | Crude hazard ratio |
| --- | --- |
| Male sex | 0.77 [0.56 - 1.08] (P = 0.13) |
| Age ≥ 70 years | 0.75 [0.55 - 1.04] (P = 0.082) |
| Non-ambulatory | 1.56 [0.92 - 2.65] (P = 0.10) |
| Smoker | 0.84 [0.61 - 1.17] (P = 0.30) |
| Type 2 diabetes | 1.09 [0.80 - 1.47] (P = 0.58) |
| Chronic kidney disease |  |
| Without dialysis | 0.81 [0.55 - 1.19] (P = 0.28) |
| On dialysis | 0.99 [0.71 - 1.40] (P = 0.97) |
| Cerebrovascular disease | 0.98 [0.65 - 1.48] (P = 0.94) |
| Coronary artery disease | 0.92 [0.68 - 1.24] (P = 0.58) |
| Chronic heart failure | 0.85 [0.56 - 1.27] (P = 0.42) |
| Atrial fibrillation | 1.31 [0.87 - 1.96] (P = 0.19) |
| Rutherford classification | 1.08 [0.90 - 1.28] (P = 0.42) |
| Ankle brachial index < 0.6 | 1.17 [0.86 - 1.58] (P = 0.32) |
| Additional aortoiliac revascularization | 1.09 [0.78 - 1.51] (P = 0.62) |
| Additional superficial femoral artery revascularization | 1.00 [0.74 - 1.35] (P = 1.00) |

Data are unadjusted hazard ratios [95% confidence intervals] (P values)
